# Dynamic Deformation Behavior of the Porcine Anterior Cruciate Ligament Enthesis Under Anterior Tibial Loading

**DOI:** 10.1007/s10439-024-03654-2

**Published:** 2024-11-27

**Authors:** Daichi Ishii, Shiho Sato, Hiromichi Fujie

**Affiliations:** Department of Mechanical Systems Engineering, Graduate School of Systems Design, Tokyo Metropolitan University, 1-1 Minami-Osawa, Hachioji-shi, Tokyo, 192-0397 Japan

**Keywords:** Anterior cruciate ligament (ACL), Enthesis, Insertion, Uncalcified fibrocartilage, Fiber orientation angle (FOA)

## Abstract

This study determined the insertion angle at the porcine anterior cruciate ligament (ACL) enthesis under joint loading to provide information on the structure and mechanical function of the enthesis. Ten intact porcine knee joints were harvested, and an anterior tibial load was applied using a robotic testing system. After dissecting a portion of the ACL enthesis along ligament fibers, the remaining enthesis was imaged using a digital microscope while reproducing the three-dimensional intact knee motion. Fiber orientation angles (FOAs) in the enthesis region (0–300 µm from the ligament-bone boundary) and the ligament region (500–2000 µm from the ligament-bone boundary) were analyzed in the femoral and tibial entheses of the anteromedial bundle (AMB) of the ACL under loading. On the femoral side, the FOA in the enthesis region was significantly higher than that in the ligament region by approximately 10 degrees under loading (*n* = 5, *p* < 0.05 in paired *t*-test). In contrast, the FOAs in the enthesis and ligament regions on the tibial side were nearly equal under loading, with no significant difference (*n* = 5, *p* > 0.15 in paired *t*-test). Histological examination indicated that uncalcified fibrocartilage (UF) was abundant in the enthesis region of the AMB femoral enthesis while the UF was not observed in the enthesis region of the AMB tibial enthesis. Thus, the current data suggest that the regional dependence and independence in FOA are caused by the presence or absence of UF and contributes to a moderate and subtle load-transduction in the ACL enthesis.

**Supplementary Information:**

The online version contains supplementary material available at 10.1007/s10439-024-03654-2.

## Introduction

The rate of injury of the anterior cruciate ligament (ACL) is higher than those of other ligaments in the knee joint [1, 2]. Reconstruction surgery is usually performed for ACL-injured knees by inserting tendon grafts through tunnels in the femur and tibia. The reconstructed ACL is sometimes re-injured at the attachment site after ACL reconstruction, indicating that the healing of the attachment site is essential [3–5]. Therefore, the native ACL attachment site (enthesis) has been investigated in many studies for a better understanding of the morphology and biomechanics of the enthesis.

Histological studies have been performed to investigate the microstructure of ligament and tendon entheses. Ligament and tendon entheses are classified into two types: fibrous entheses, in which ligaments and tendons attach directly to bone via the periosteum, and fibrocartilaginous entheses, in which ligaments and tendons attach indirectly to bone via non-calcified fibrocartilage (UF) and calcified fibrocartilage (CF) [6–12]. Generally, the fibrous enthesis is observed in long bone diaphysis, while fibrocartilaginous enthesis is observed in epiphyseal or apophyseal long bone ends. In contrast to ligaments and tendons that mainly contain type I collagen, UF and CF of the fibrocartilaginous enthesis are rich in type II collagen and proteoglycans, and CF also contains type X collagen. In the ACL attachment site, fibrous enthesis and fibrocartilaginous enthesis are sometimes referred to as direct and indirect insertions, respectively [13–16]. Iwahashi et al. and Mochizuki et al. found that the human ACL femoral enthesis contains both direct and indirect insertions [13, 15]. On the other hand, Beaulieu et al. described that the femoral and tibial entheses of the human ACL are fibrocartilaginous tissues and the area of fibrocartilage is larger in the femur compared to the tibia [17, 18].

Histological studies of the ligament/tendon enthesis estimated the mechanical functions of the enthesis. For example, fibrocartilage is hypothesized to play a role in gradually changing the modulus from ligament to bone like a functionally graded material, and that UF plays a role in reducing the acute insertion angle of the ligament like the grommet of an electric plug, preventing injury at the enthesis [6, 10, 11]. However, the previously reported estimations are not supported with quantitative data. Sevick et al. observed the deformations of the medial collateral ligament (MCL) femoral enthesis and Achilles tendon enthesis of rabbits under tensile loading and found that the insertion angle between UF and CF decreased under loading [19]. Sartori et al. also observed a similar angular change in Achilles tendon enthesis of rats in an ex vivo examination [20]. Quantitative investigation on the angle of the soft tissue of UF relative to the hard tissue of CF under loading is important, as in the previous studies, to confirm the estimation on the mechanical function of the enthesis that UF moderates the acute insertion angle of ligament. However, it is also important to investigate not only the angle between the CF and the UF, but also the angle between the CF and the ACL, and to compare the angle of UF and ACL. In addition, the mechanical environments around the MCL and Achilles tendon entheses of small animals such as rats and rabbits are expected to be different from that around the ACL entheses of large animals such as humans and pigs.

Therefore, in this study, the dynamic deformation behavior of the porcine ACL enthesis under joint loading was determined using a robotic joint testing system, with a special focus on the fiber orientation angle (FOA) between the hard and the soft tissue of the UF and ligament. The objective of this study was to quantify the FOA of UF and ligament midsubstance in the porcine ACL enthesis and evaluate differences between FOAs. The hypothesis was that the UF moderates the acute insertion angle of the ligament fiber under physiological loading in the ACL enthesis.

## Materials and Methods

Porcine knee joints (*n* = 10) were harvested from 6-month-old 3-crossbred pigs. Five knees were randomly selected and used to observe the deformation behavior of the femoral enthesis, and the remaining five knees were used to observe that of the tibial enthesis. All knee joints were stored at − 30 °C and transferred to 4 °C one day before examination. Fat, muscle, and the patella were removed with care, ensuring not to damage the ligaments, meniscus, and posterior joint capsule. The femur and tibia were cut at approximately 150 mm from the joint line and reinforced with a polymethyl methacrylate bone cement (OSTRONII, GC Corporation, Tokyo, Japan) for fixation to the robotic system.

**Fig. 1:**
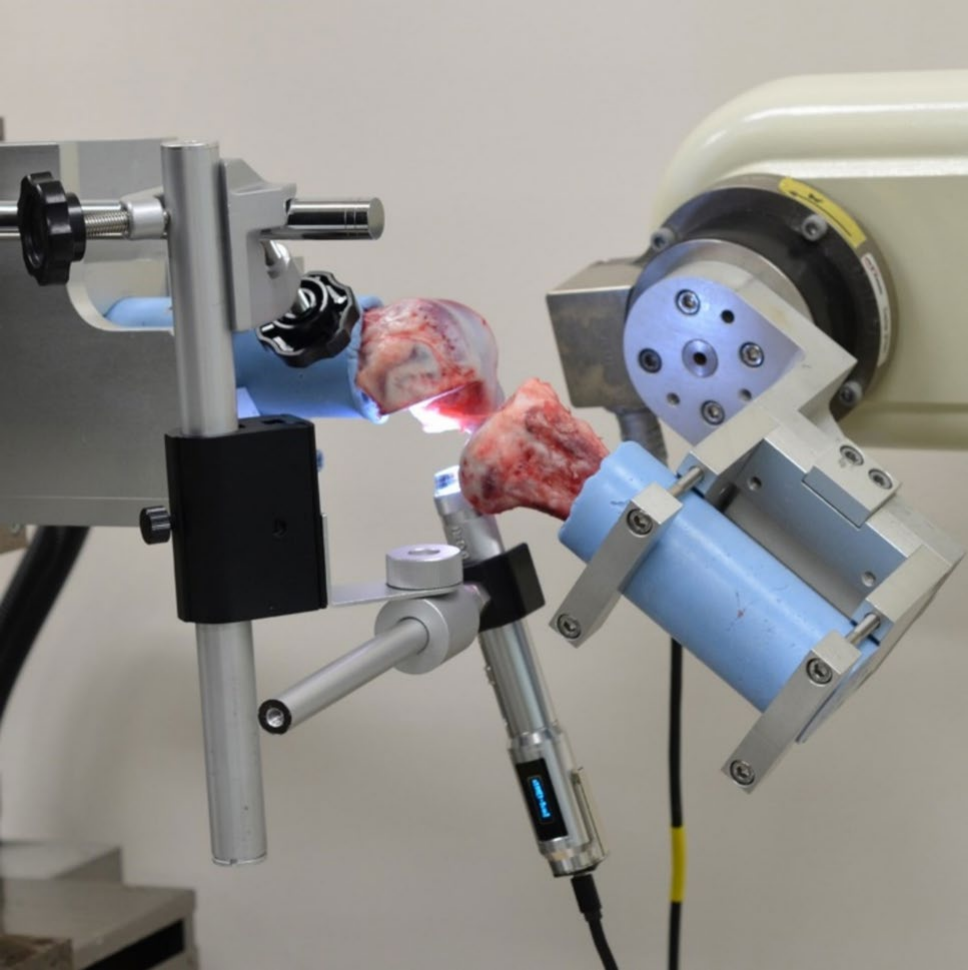
A porcine knee joint and the digital microscope fixed to the 6-DOF robotic system (In this figure, deformation of the femoral enthesis was captured)

**Fig. 2:**
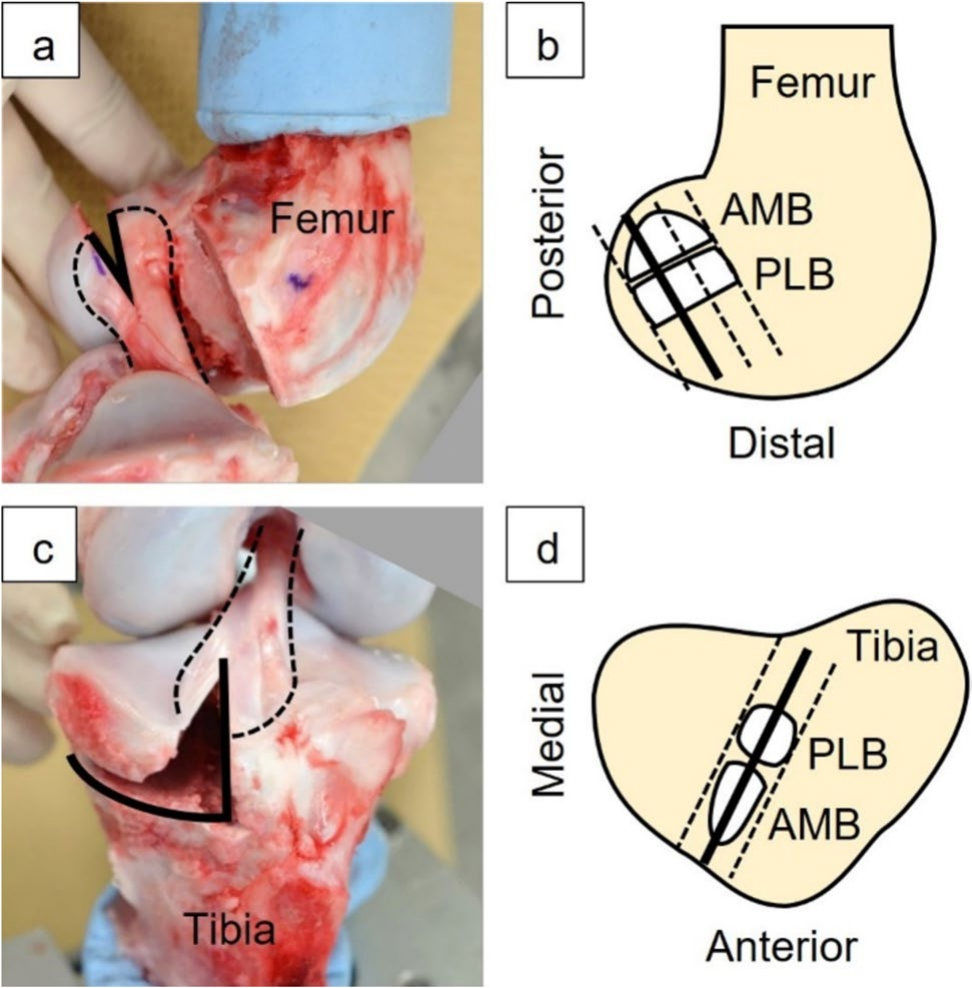
Preparation of the cross section in the femur (**a, b**) and tibia (**c, d**). The femoral lateral condyle and tibial medial condyle of the porcine left knee joint were removed at defined positions (black line) to reveal the enthesis

A 6-DOF robotic system (FRS-2015, Technology Services, Nagano, Japan) was used for the following tests [21]. The robotic system consists of a custom made 6-axis manipulator and a 6-DOF universal force/moment sensor and enables simulation of knee joint motion based on the knee joint coordinate system using position and force control [22–28]. An intact porcine knee joint was fixed to the clamps of the robotic system (Fig. 1). Three knee positions were selected: 1) full extension, 2) 60° and 3) 90° of flexion. The full extension of the porcine knee joint is approximately 30° and does not reach 0° as in humans [29]. The full extension position was defined by applying a flexion-extension moment of 1 Nm to the knee while controlling the forces and moments along and about the other five axes at 0 N and 0 Nm [30, 31]. Flexion positions of 60° and 90° were defined as the positions where the flexion-extension rotation angle was 60° and 90°, respectively, while controlling the forces and moments along and about the other five axes at 0 N and 0 Nm. At each flexion angle, anterior tibial forces were applied up to 200 N at a rate of 0.2 mm/s. At this time, three-dimensional motion of the robotic system was recorded during the test. After the test on the intact state, the knee joint and clamps were removed from the robotic system, and the medial femoral condyle and soft tissues except for the ACL were dissected. The cross sections of the ACL femoral and tibial entheses were prepared by removing part of the lateral femoral condyle and part of the tibial condyle, respectively (Fig. 2). The ACL fibers attached to the removed bones were peeled off from the remaining ACL and cut at the midsubstance about 15 mm from the attachment site. Special care was taken not to damage the fibers during this peeling. Both cross sections were parallel to the direction of the ACL fibers in full extension and perpendicular to the surface of the ACL attachment site (Fig. 2). The cross section of the femoral enthesis was positioned one-third posterior to the attachment site, and the cross section of the tibial enthesis was positioned at the half width of the attachment site. The positions of these cross sections were determined based on a preliminary examination. Finally, we confirmed that the fibers of the enthesis could be clearly observed in these cross sections.

After the dissection, the knee joint and clamps were re-fixed to the robotic system. Note that the coordinate system of the knee joint and robot system was maintained during the removal and re-fixation of the knee joint and clamps [32–36]. A digital microscope (3R-MSBTVTY, 3R Solution, Fukuoka, Japan) was then fixed to the clamps using a custom-made holder (Fig. 1). The microscope was adjusted in such a position that the imaging direction was perpendicular to the cross-sectional surfaces prepared in the dissection. The dynamic deformation behavior of the ACL enthesis was captured during the reproduction of the three-dimensional motion recorded in the anterior tibial loading test of the intact knee. The dynamic deformation behavior of the enthesis was captured again after graphite particles of approximately 5 µm in diameter were scattered on the cross sections of the ACL entheses. After imaging, the remaining ACL connecting to the femur and tibia was cut and the reproduction of intact joint motions was repeated. Based on the difference in the force/moment sensor output before and after the ACL was cut, the in situ force in the ACL (ACL force) at the time of imaging was calculated based on the principle of superposition [37].

**Fig. 3:**
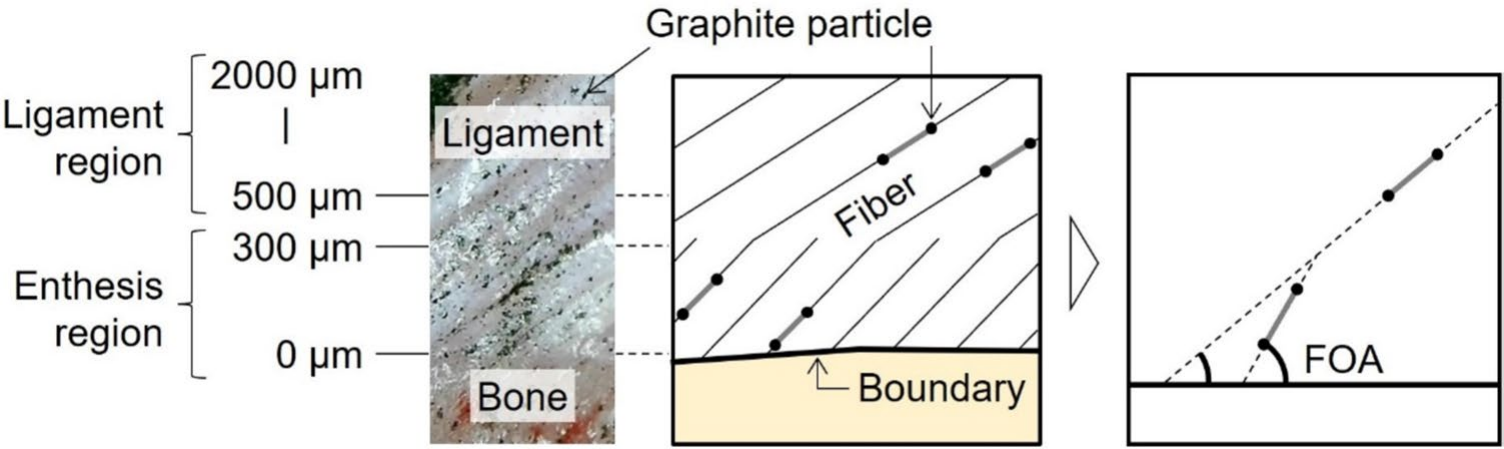
Quantitative analysis method of fiber orientation angles (FOAs) and definition of regions. Graphite particles parallel to the fiber direction were tracked in each region, and the FOA between this line segment and the approximate line of the ligament-bone boundary was calculated

FOA was quantitatively analyzed on the enlarged image of the anteromedial bundle (AMB) in the femoral and tibial entheses when the surface of the ligament and bone stayed in the original cross section of the ACL enthesis. This was confirmed by in focus imaging of the cross section under anterior tibial loading. The AMB and posterolateral bundle (PLB) of the ACL were determined with reference to previous studies and preliminary experiments (Fig. 2) [38–40]. For this analysis, an image analysis software (Motion Analyzer VW-H2MA Ver. 1.5.0.0, Keyence, Osaka, Japan) was used, which can track arbitrarily selected points on the moving image based on the image correlation method. In the deformation behavior of AMB enthesis coated with graphite particles, the area of 0–300 µm from the ligament-bone boundary to the ligament was defined as the enthesis region, and the area of 500–2000 µm was defined as the ligament region (Fig. 3). The movement of the particles under anterior tibial loading was tracked using the software. Three to six pairs of graphite particles were selected in each region, so that a pair of graphite particles of approximately 100 µm apart were located parallel to a fiber. The angle between the straight line connecting the paired graphite particles and the approximate straight line at the ligament-bone boundary was calculated. The mean value was determined as the FOA for each region.

Paired *t*-tests were performed to examine the differences in FOA between the ligament and enthesis regions, and the one-way repeated measures ANOVA was performed to examine the changes in FOA with increasing anterior tibial load. The significance level of each test was set at *p* < 0.05. Statistical analysis software (IBM SPSS Statistics Ver. 22.0.0.0, International Business Machines Corporation, USA) was used for these analyses.

Finally, the porcine knee joints (*n* = 5) that were used to histologically evaluate the fibrocartilage within the enthesis were evaluated. The knee joints were fixed by soaking the specimens in formalin for 48 hours in the full extension. After fixation, the ACL and its attachment site were revealed, and a cross-sectional sample was prepared in the same manner as for the deformation behavior analyses. The samples were dewaxed, demineralized, dehydrated, embedded in paraffin, sliced to 6 µm, and stained with toluidine blue to evaluate fibrocartilage areas [41].

## Results

**Fig. 4:**
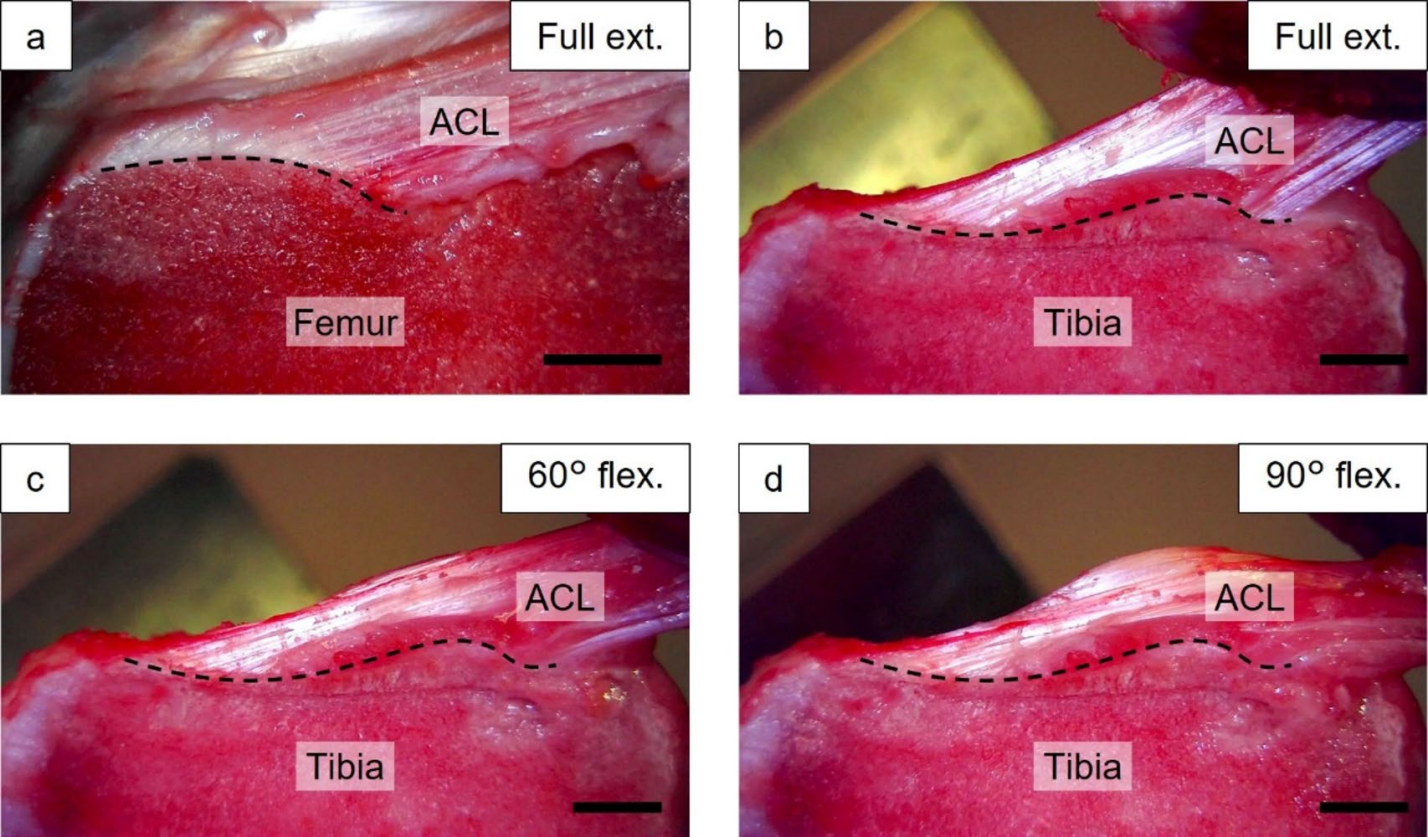
Typical deformation behavior of the ACL femoral (**a**) and tibial (**b–d**) enthesis under an anterior tibial force of 200 N. The fiber orientation angle of the entire ACL relative to the ligament-bone boundary (dotted line) decreased with increasing flexion angle at tibial enthesis. Scale bar = 5 mm

**Fig. 5:**
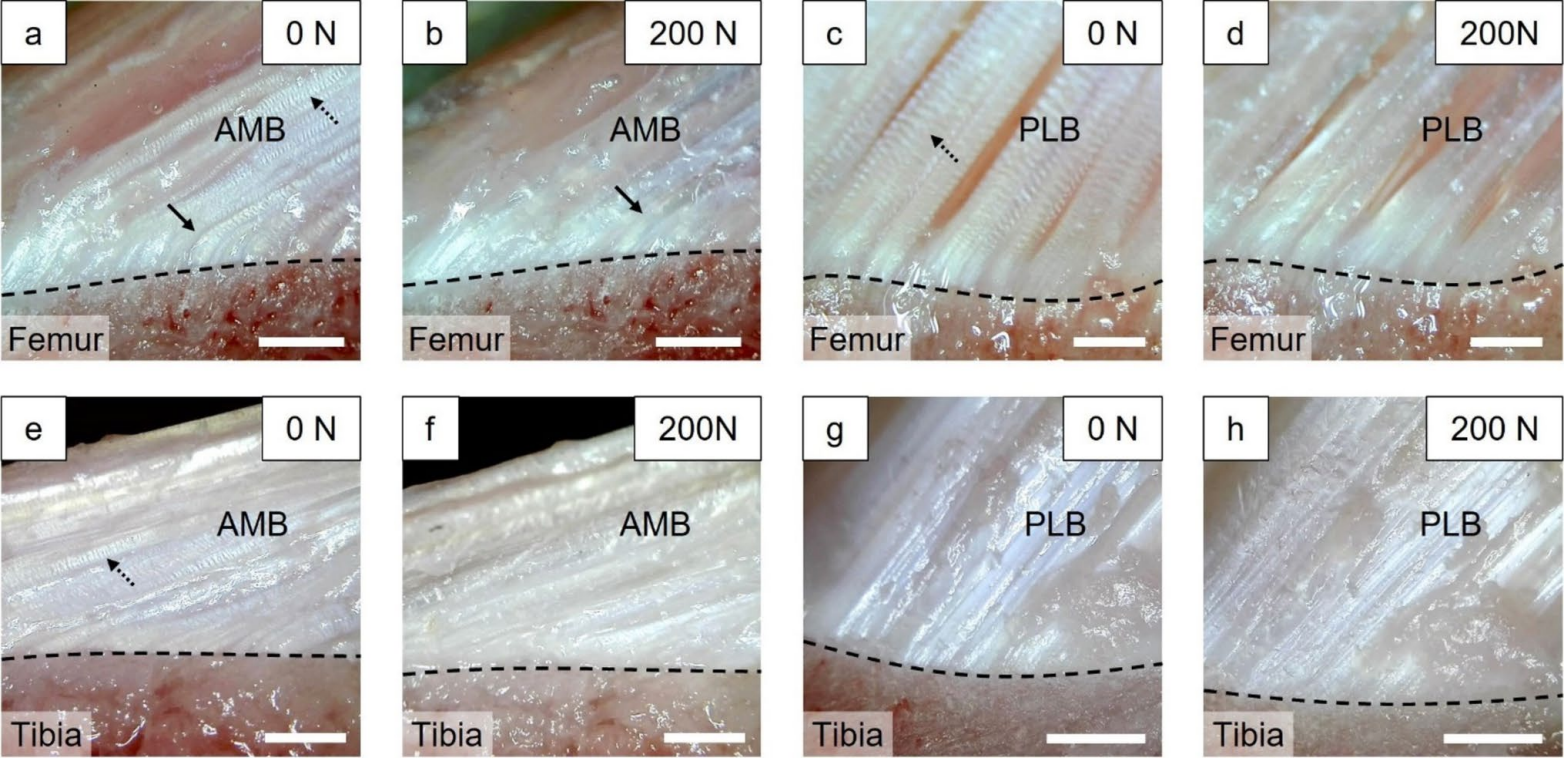
Typical enlarged deformation behavior of the femoral (**a–d**) and tibial (**e–h**) entheses under an anterior tibial loading of 0, 200 N at full extension. The disappearance of crimps was observed in the AMB and the PLB femoral entheses and in the AMB tibial enthesis (dotted arrows in a, c, and e). The maintaining of fiber flexion under anterior tibial loading was observed in the AMB femoral enthesis (arrows in a, b). Scale bar = 500 µm

The captured image of the femoral enthesis was in focus at full extension (Fig. 4a), but out of focus at 60° and 90° flexion. Conversely, the captured image of the tibial enthesis was in focus at full extension, 60° and 90° flexion (Fig. 4b–d). In all deformation states, the ACL was elongated with increasing anterior tibial load. In the tibial enthesis, the FOA of the ACL fibers decreased with the increasing flexion angle. Enlarged images of each fiber bundle enthesis were captured only under loading at full extension (Fig. 5). Some enthesis showed the disappearance of the crimp structures in the ligament substance with the increase in anterior tibial load. Fibers in the AMB femoral enthesis at full extension were always flexed in the boundary area between the UF and ligament substance despite loading, while fibers in the AMB tibial enthesis and the PLB femoral and tibial enthesis were always straight. The dynamic deformation behaviors of the ACL entheses described above can be viewed in the videos (Online supplementary resources 1–8). These videos show the dynamic deformation behaviors of the ACL entheses under 0–200 N of anterior tibial loading, with playback speed adjusted to approximately 5x the original speed.

After creation of the femoral enthesis cross section, the tensile force applied to the remaining ACL was 93 ± 18 N and 193 ± 20 N under 100 and 200 N of anterior tibial force, respectively, in full extension. The tensile force applied to the remaining ACL after creation of the tibial enthesis cross section was 72 ± 19 N and 152 ± 28 N under 100 and 200 N of anterior tibial force, respectively, in full extension. In both cases, the tensile forces increased linearly with increasing anterior tibial load.

**Fig. 6:**
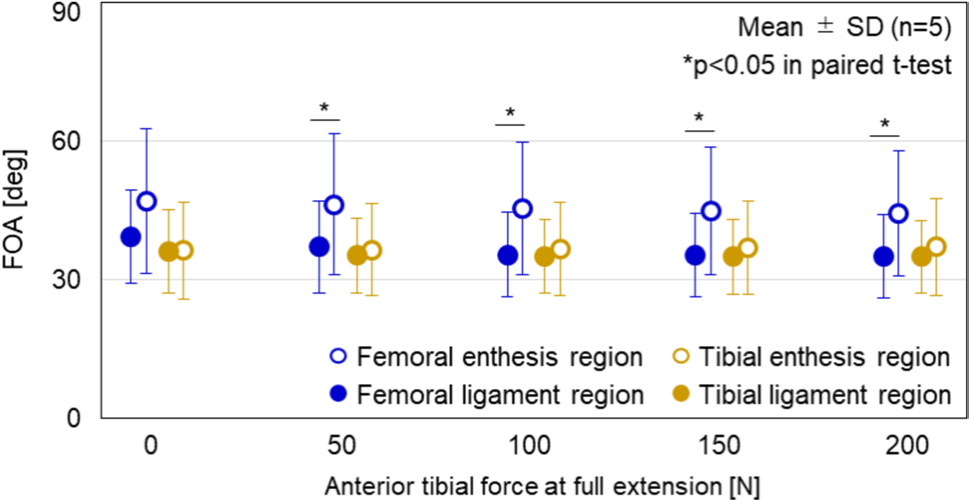
Quantitative analysis of fiber orientation angle (FOA). Each point and error bar indicate the mean and the standard deviation, respectively. The FOA of the enthesis region was significantly larger than that of the ligament region under 50, 100, 150, 200 N of anterior tibial loading at full extension in the AMB femoral enthesis (*p* < 0.05 in paired *t*-test). There was no significant decrease in FOA with increasing anterior tibial load in all regions (*p* > 0.05 in one-way repeated measures ANOVA)

The AMB femoral enthesis indicated that the FOAs of the enthesis and ligament regions were 47.0 ± 15.7° and 39.3 ± 10.0°, respectively, at no load, and 44.4 ± 13.5° and 35.1 ± 9.1°, respectively, at 200 N of anterior tibial force in full extension (Fig. 6). The FOA in the enthesis region was significantly larger than that in the ligament region under 50, 100, 150, and 200 N of anterior tibial force (*p* = 0.032, 0.038, 0.031, 0.025, respectively). In addition, there was no significant change in FOA with increasing load in the enthesis region (*p* = 0.207) and the ligament region (*p* = 0.058). The FOAs of the AMB tibial enthesis were approximately 35 ± 10° in both regions under 0, 50, 100, 150, and 200 N of anterior tibial loading in full extension (Fig. 6). There was no significant difference between regions at any loading (*p* > 0.15), and no significant change in FOA with increasing anterior tibial load (*p* = 0.337 in the enthesis region, *p* = 0.360 in the ligament region).

**Fig. 7:**
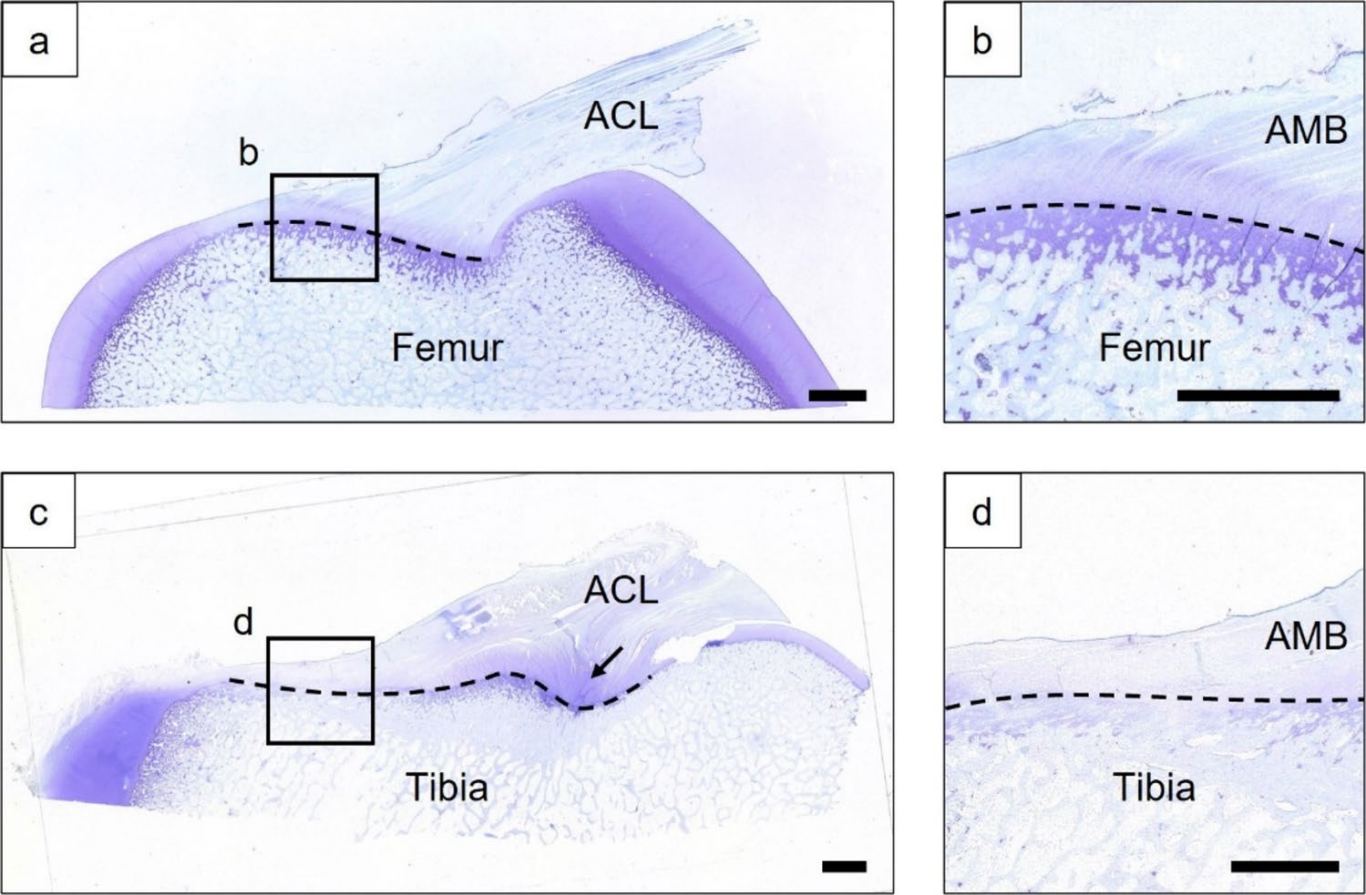
Typical toluidine blue stained images of the femoral (**a, b**) and tibial (**c, d**) entheses. Uncalcified fibrocartilage (UF) and calcified fibrocartilage (CF) were observed in the ACL femoral enthesis (**a**), and in the anteromedial bundle (AMB) femoral enthesis (**b**), UF was observed on the soft tissue side above the tidemark (dotted line). In the ACL tibial enthesis (**c**), the fibrocartilaginous area was observed in the PLB enthesis (arrow), and UF was not observed in the AMB enthesis (**d**). Scale bar = 2 mm

The results of toluidine blue staining showed that the UF and CF regions were approximately 500 µm in thickness in the femoral enthesis (Fig. 7a, b). In the tibial enthesis, rich UF was found in the PLB enthesis, while UF was not found in the AMB enthesis (Fig. 7c, d).

## Discussion

The results of the histological examination were similar to previous studies on the porcine ACL enthesis [40, 42]. However, in this study, the histological structure of the ACL enthesis and its dynamic deformation behaviors were observed. The histological examination indicated that the ligament-bone boundary observed in the mechanical test corresponded to the tidemark between the UF and CF (Figs. 4, 5, and 7). This finding confirmed that the enthesis region defined in the FOA analysis was the UF region in the AMB femoral enthesis and ligament region in the AMB tibial enthesis.

**Fig. 8:**
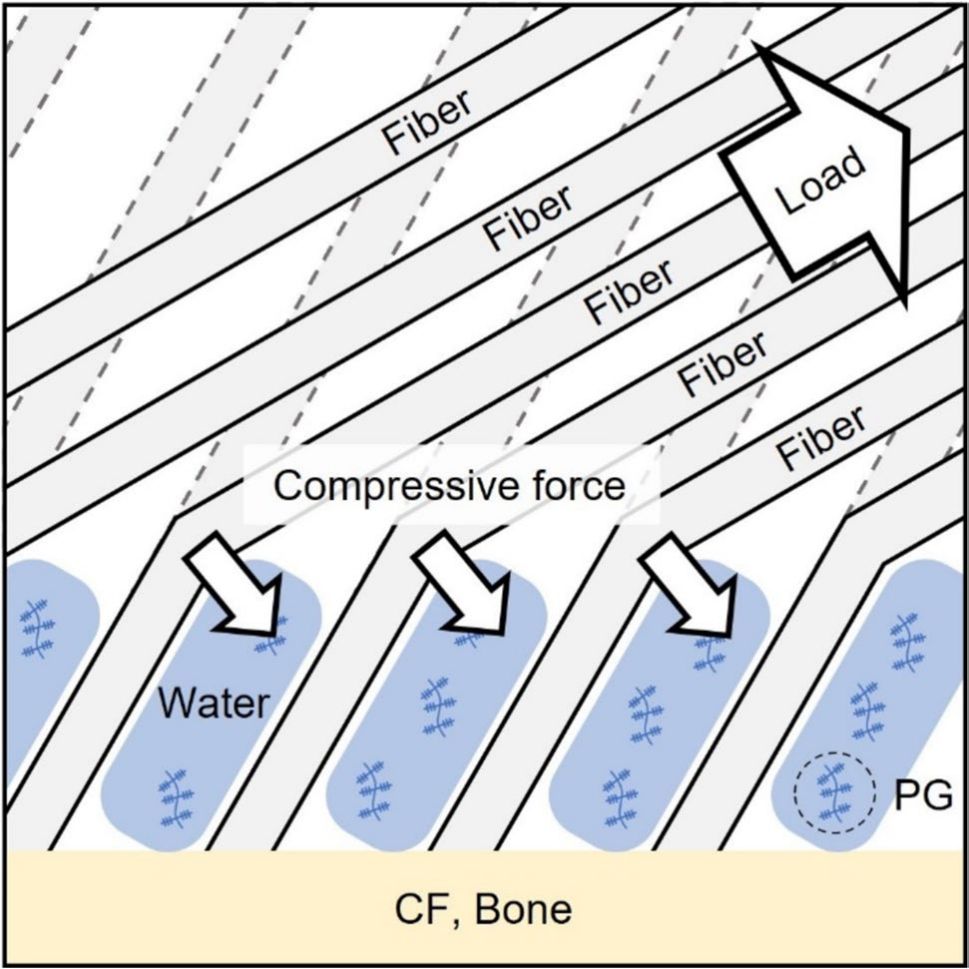
Schematic diagram of compressive forces applied to UF. Under loading, ACL fibers bend due to the UF, which contains proteoglycans (PG) with high water content. This bending generates compressive forces to the UF, CF, and bone

The FOA in the enthesis region was greater than that in the ligament region in the AMB femoral enthesis, with significant differences observed under anterior tibial loading (Fig. 6). In contrast, there was no significant difference of FOA in the tibial enthesis regardless of anterior tibial load. For both the femoral and tibial entheses, deformations of the AMB enthesis used for FOA analysis were caused by anterior tibial loading at full extension. Furthermore, during the capture of these deformations, the ACL forces were almost equivalent, and the FOA of the ligament region was approximately 35° in both entheses. Therefore, the fiber bending angle (= FOA difference) in the AMB femoral enthesis may be attributed to the UF. Previous studies reported that the UF plays a role in resisting compressive force due to the proteoglycans with high water content in the UF [8–10, 43, 44]. In this study, the FOA of the UF was larger than that of the ligament in the AMB femoral enthesis, likely due to the resistive property of UF to compressive forces (Fig. 8). On the other hand, in the femoral and tibial enthesis of PLBs with UF, UF fibers, and ligament fibers were oriented straight without bending (Figs. 5 and 7). This result may be due to the large insertion angle of the ligament fibers. Therefore, the function of UF to moderate the insertion angle of the ligament fibers was only demonstrated when the insertion angle of the ligament fibers was low. Fiber flexion in the MCL enthesis due to the small FOA of the ligament was also observed by Thambyah et al. in which the structural observation of the bovine MCL enthesis was performed under tensile force [45]. Furthermore, the UF has traditionally been hypothesized to alter the insertion angle at the microstructural level, similar to the function of grommets in electric plugs, to prevent injury at the enthesis, based on structural observations [6, 8, 11]. Therefore, the findings of the current study provide novel quantitative data in the ACL enthesis under the physiological loading, supporting the theory that the UF prevents injury at the enthesis.

In the current study, FOAs of the enthesis and the ligament region tended to decrease with increasing anterior tibial load from 0 to 200 N at the AMB femoral enthesis although the difference was not statistically significant. Sevick et al. and Sartori et al. reported that the angle of the UF was decreased with increasing load in the rabbit MCL enthesis and Achilles tendon enthesis, and the rat Achilles tendon enthesis, although they did not perform statistical analysis [19, 20]. Therefore, this trend found in this study is consistent with those obtained in previous studies. However, the current study revealed that the difference between the FOA of the ligament region and that of the enthesis region was maintained even though the FOA of both regions decreased with increasing anterior tibial load.

A novel aspect of the current study was observation and quantification of the dynamic deformation of the ACL enthesis under joint loading. However, this study has several limitations. Firstly, the ACL enthesis, including ligament fibers and bones, were transected to create the cross section for observation, which might have influenced the behavior of microstructures in the enthesis. However, clear observation of the cross-sectioned ACL enthesis and adjacent bone was possible under loading by preparing cross sections parallel to the direction of the ACL fibers, which implies that the native enthesis construct was maintained. In addition, a large tensile force close to anterior tibial force was applied to the remaining ACL tissue, and the crimp pattern disappeared in the cross section with the increasing anterior tibial load as observed elsewhere (Fig. 5) [46–48]. Therefore, we believe that tissue transection had minimal effect on the experimental results. Secondly, there were limitations in the sample and test conditions. While the anatomy and mechanical function of porcine knees and ACLs are more similar to those of humans compared to other animals, there are some differences such as the location of AMB and PLB attachments and the angle of full extension [29, 38, 40, 49–54]. Additionally, in FOA quantitative analysis, only anterior tibial force was applied to the ACL enthesis at full extension. It is expected that the results would be different under test conditions in flexion. Therefore, it is necessary to observe and analyze similar dynamic behavior in human ACL entheses under the anterior tibial loading at multiple flexion angles in future studies.

In conclusion, the dynamic deformation behavior of the ACL enthesis under anterior tibial loading was analyzed. It was found that UF can moderate acute insertion angle of ACL fibers under loading, with variations depending on the physiological load and the entheses of each bundle. This finding will provide detailed insights into the mechanical function of the enthesis, which is crucial for the consideration of appropriate clinical treatments and joint function.

## Supplementary Information

Below is the link to the electronic supplementary material.

Supplementary file1 (MP4 14425 KB)

Supplementary file1 (MP4 14720 KB)

Supplementary file1 (MP4 14457 KB)

Supplementary file1 (MP4 15006 KB)

Supplementary file1 (MP4 14308 KB)

Supplementary file1 (MP4 14417 KB)

Supplementary file1 (MP4 13750 KB)

Supplementary file1 (MP4 14953 KB)

## Supplementary Information

Below is the link to the electronic supplementary material.
